# Procedural characteristics and acute outcomes with different pulsed field ablation systems for atrial fibrillation

**DOI:** 10.1007/s10840-026-02389-5

**Published:** 2026-06-27

**Authors:** Francis J. Ha, Hui-Chen Han, Adam J. Brown, Nitesh Nerlekar, Emily Kotschet

**Affiliations:** Victorian Heart Institute and Victorian Heart Hospital, Monash University, Monash Health, 631 Blackburn Road, Clayton, VIC 3168 Australia

**Keywords:** Atrial fibrillation, Pulsed field ablation

## Abstract

**Background:**

Multiple different pulsed field ablation (PFA) systems have been developed for the treatment of atrial fibrillation (AF). An improved understanding of procedural workflow and outcomes are needed.

**Methods:**

We performed a comparative observational study of consecutive patients undergoing PFA with three systems at a tertiary centre: Farawave without electroanatomic mapping (Boston Scientific, US), Faraview with integrated electroanatomic mapping (Boston Scientific, US) and Varipulse (Johnson and Johnson MedTech, US). All patients underwent pulmonary vein isolation (PVI) with additional lesions at operator discretion.

**Results:**

A total of 150 patients undergoing PFA were included (50 with each system). Median age was 63–65 years (*p* = 0.28) and 28–38% female (*p* = 0.60). Persistent AF ranged from 40 to 62% (*p* = 0.09). More patients received posterior wall (PW) ablation with Faraview (50%) compared with Varipulse (26%) and Farawave (24%) (*p* = 0.01). Faraview was associated with more PV applications (52 versus 36; *p* < 0.001) although fewer PW applications (18 versus 24; *p* = 0.028) compared with Farawave. Procedure and left atrial catheter dwell times were higher with Varipulse compared with Farawave and Faraview (*p* < 0.001), but fluoroscopy time was lower (*p* < 0.001). Fluoroscopy use with Varipulse reduced in the second 25 cases compared with the first 25 cases (*p* = 0.01). There was no difference in rate of major or minor adverse events between groups.

**Conclusions:**

These real-world data on different PFA systems highlight varying procedural workflows depending on catheter design and the availability of additional information such as electroanatomic mapping and tissue contact. Further randomised studies are needed to compare safety and efficacy of different PFA systems for AF.

**Graphical abstract:**

**Figure:**
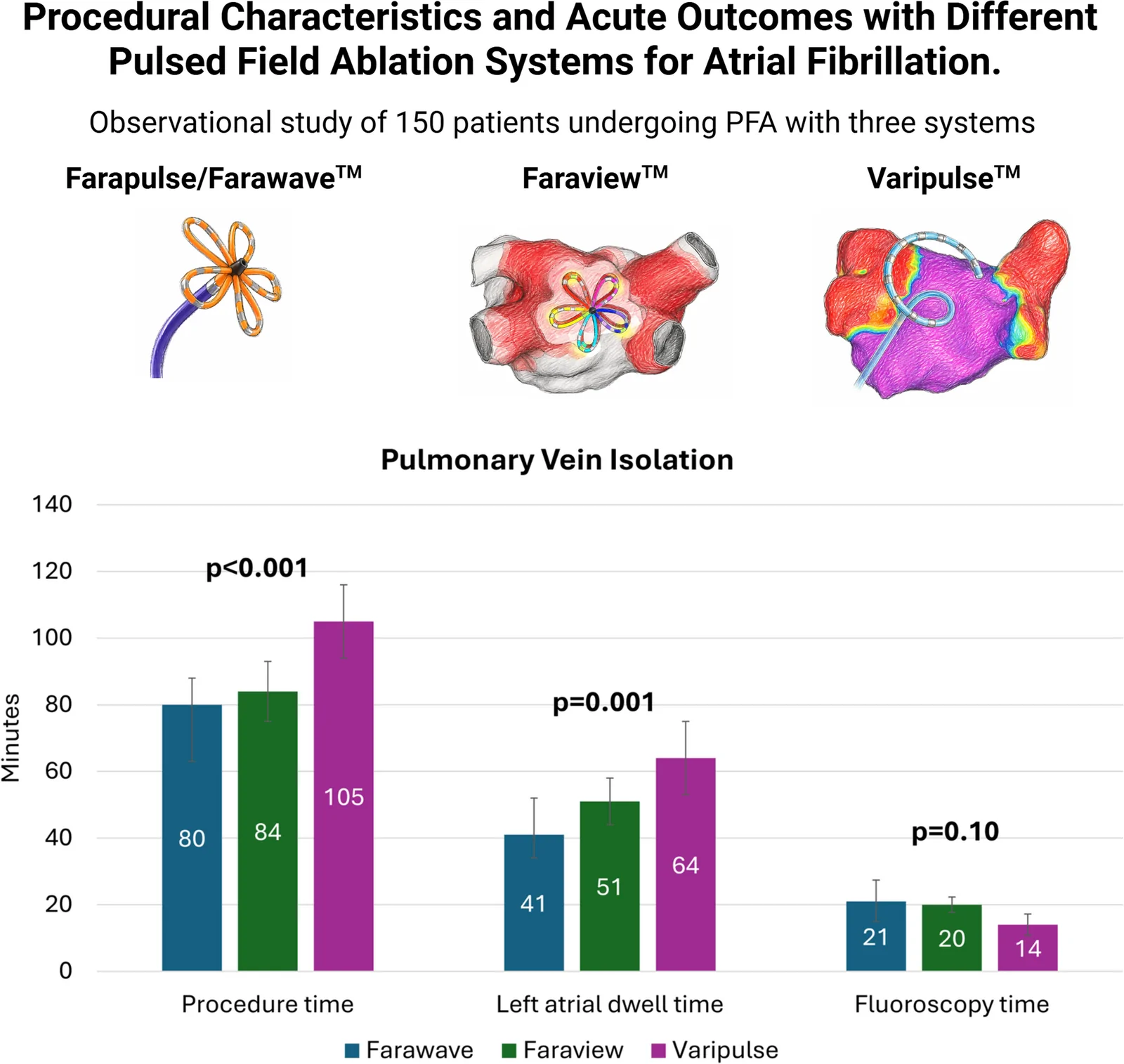

## Introduction

**Fig. 1:**
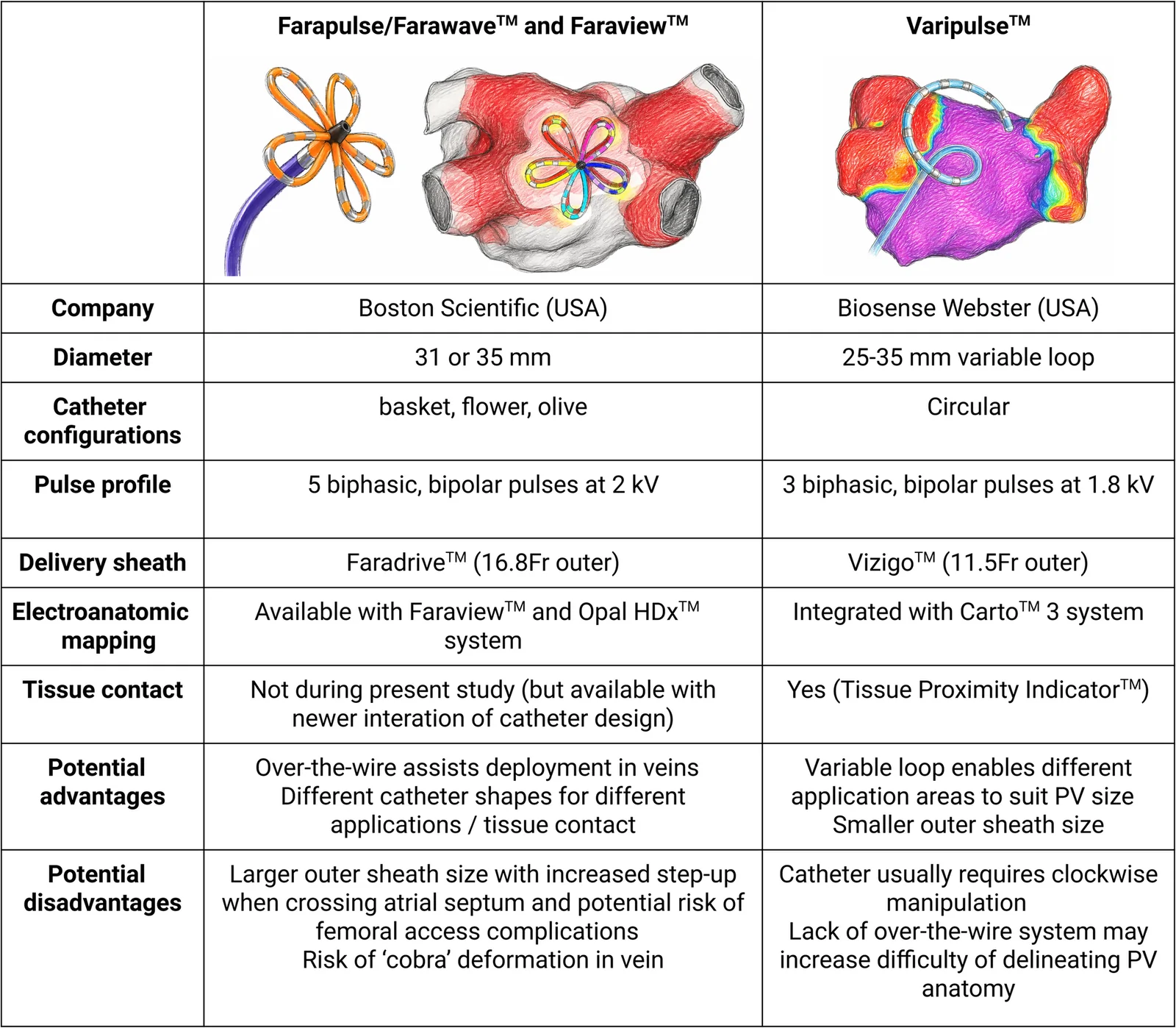
Catheter design of different PFA platforms. *PV, Pulmonary vein*

Pulsed field ablation (PFA) has emerged as a leading form of catheter ablation for atrial fibrillation (AF) [1]. Coupled with improved procedural workflow compared with conventional thermal ablation, it tends to be more selective for cardiac tissue and so far avoids concern for atrio-oesophageal fistula [2, 3]. Numerous PFA systems have become commercially available over the last several years, each with different catheter shapes, profiles and delivery of pulsed field application. Farapulse/Farawave^™^ (Boston Scientific, USA; referred to as Farawave onwards) has been investigated in clinical trials and large post-approval registry data with similar efficacy to thermal ablation and acceptable safety profile [1, 4, 5]. It is a large footprint, multielectrode pentaspline catheter with an over-the-wire profile manipulated through a deflectable sheath with various catheter shapes from ‘flower’ and ‘basket’ conformations. Farawave Nav^™^ with Faraview^™^ (referred to as Faraview from here onwards) is a later iteration of Farapulse which incorporates electroanatomic mapping (EAM) for geometry and Fieldtags^™^ (Boston Scientific) to show areas of ablation and guide degree of lesion overlap. Varipulse^™^ (Biosense Webster, USA) is another PFA system that uses an 8.5 Fr bidirectional variable loop circular catheter (VLCC) with ten 3 mm electrodes and three magnetic, single axis sensors through an outer sheath in combination with EAM and software enabling information about catheter-tissue impedance-based contact force (Tissue Proximity Indicator^™^, Biosense Webster, USA) [6, 7]. A comparison of Farapulse/Faraview and Varipulse catheter design including potential advantages and disadvantages are shown in Fig. 1.

Limited real-world clinical data exist comparing these PFA systems in terms of procedural workflow and procedural outcomes. We aimed to evaluate patient demographics, procedural characteristics and procedural outcomes between the Farawave system, Faraview system, and the Varipulse system.

## Methods

We performed a retrospective observational study of consecutive patients who underwent PFA for paroxysmal or persistent AF at a tertiary centre with seven operators from November 2022 (initial commencement of PFA) to November 2025. The first 50 consecutive patients undergoing de novo PFA using each catheter system – Farawave (November 2022 to May 2023), Faraview (March to June 2025), and Varipulse (July to October 2025) – were included. All operators were familiar with manipulation of larger diameter catheters in the left atrium from previous cryoballoon ablation experience. The initial 50 consecutive patients from the collective experience of across operators with each PFA system were obtained for this study. Approval for this study was granted by the local institution’s Human Research Ethics Committee.

### Baseline demographics

Clinical data were obtained from electronic medical records. Baseline demographic variables included age, sex, body mass index (BMI), atrial fibrillation (AF) type (paroxysmal or persistent), and relevant medical comorbidities. The CHA₂DS₂-VASc score was calculated for each patient. Renal function was evaluated using the estimated glomerular filtration rate (eGFR), and left ventricular ejection fraction (LVEF) and indexed left atrial volume (LAVI) were recorded when available. AF type was classified as paroxysmal, defined as self-terminating episodes lasting less than seven days, or persistent, defined as sustained episodes lasting seven days or longer. Medication data included antiarrhythmic and anticoagulant therapies prescribed prior to the ablation procedure.

### Pulsed field ablation procedure

All patients underwent PFA under general anaesthesia. Pulmonary venous anatomy and left atrial appendage assessment was performed using transesophageal echocardiography (TOE) or contrast venography at operator discretion during the procedure. Periprocedural anticoagulation strategy was according to physician preference however the most common practice at our centre is either uninterrupted anticoagulation or omission of the oral dose on the morning of the procedure with resumption of anticoagulation the night of the procedure if there is groin haemostasis. Femoral venous access was obtained for each patient with a 7Fr and 8Fr sheath guided by ultrasonography. A multipolar catheter was inserted into the coronary sinus for the duration of the procedure. If patients were in AF at the beginning of the procedure, they were routinely cardioverted prior to performing the transseptal puncture. Transseptal puncture was performed with a Swartz SL1 8.5Fr sheath under TOE and hemodynamic monitoring guidance. Peri-procedural intravenous heparin was administered to achieve a target activated clotting time (ACT) of 350 s or above. Atropine was administered to minimise the risk of vagal response.

### Farawave

Following transseptal puncture, a J-tipped wire was typically advanced into the left superior PV to facilitate sheath cannulation directly with the Faradrive sheath to left upper PV, or after the wire and dilator was removed, an Amplatz Extra Stiff guidewire (Cook Medical, USA) was used to wire the LSPV and the Faradrive deflectable 13 F sheath passed over it. The Farawave catheter was then introduced through the Faradrive sheath about 15 cm with an initial aspiration and flush through the three-way intravenous line on the Faradrive sheath using a 50mL luer lock syringe with careful manual inspection for any air entrainment. Either a 31–35 mm pentaspline catheter was selected based on left atrial size. Each PV received at least 4 applications in ‘flower’ and ‘basket’ configuration at 2 kV with paired lesions over a Rosen wire (Cook Medical, US). PW ablation was performed with overlapping applications across the PW covering both the superior and inferior aspect with isolation confirmed by loss of PW electrograms (EGMs) after ablation. Additional lesions were applied at operator discretion where there were perceived gaps after initial lesion set guided by fluoroscopy and local intracardiac EGMs.

### Faraview

The Farawave Nav catheter was advanced through the Faradrive sheath over a J-tipped wire usually into the left superior PV with the pentaspline catheter opened in the body of the left atrium for initial baseline visualization on the Farawave / Opal HDx^™^ Mapping System (Boston Scientific, USA). An initial electroanatomic map was created for geometry and bipolar voltage measurements of the left atrium and pulmonary veins in combination with the automated Fieldtag^™^ marker to guide extent of PFA lesion sets and degree of overlap after each application. After PFA, a repeat electroanatomic map was performed to assess for any potentially missed areas and further applications were performed according to operator discretion.

### Varipulse

Following transseptal puncture, a J-tipped wire was typically advanced into the left superior PV to facilitate sheath cannulation with the SL1 sheath. This was then exchanged directly with a Vizigo^™^ deflectable sheath (Biosense Webster, USA). The VLCC was then introduced in a neutral undeflected, maximal diameter loop position through the sheath about 15 cm with an initial aspiration and flush through the three-way intravenous line on the sheath using a 20mL luer lock syringe with careful manual inspection for any air entrainment. Initial EAM was performed using the VLCC catheter using the Carto^™^ 3 system (Biosense Webster, USA). The typical workflow for ablation of PVs entailed four full ablations per vein to create two concentric rings in an ostial and then antral fashion around the PV sleeves. A combination of both sheath deflection and catheter D-curve deflection was sometimes used to achieve adequate vein apposition and coverage. Contact was confirmed using Tissue Proximity Indicator^™^ (TPI) on the Carto system. After each ablation the catheter was rotated to cover the lesion gap between electrodes 1 and 10. The generator (Truepulse^™^; Biosense Webster, US) was programmed to deliver three applications per ablation. The flow rate of heparinised saline through the ablation catheter was adjusted from 4mL/min when not ablation to 30mL/min during ablation. A post-ablation electroanatomic map was performed with the VLCC to assess for any potentially missed areas and further applications were performed according to operator discretion.

### Ablation of non-PV triggers and procedural end point

PFA was typically performed on the PVs in all cases, and ablation of the posterior wall (PW) and other non-PV triggers varied according to physician preference. Procedural end point for isolation of PVs and PW was the loss of local EGM signals after ablation. Assessment of entrance and exit block was at operator discretion although was not routinely performed.

### Post-ablation

Upon completion of PFA, catheters were removed and femoral closure performed with a figure-of-eight suture. All patients were monitored in the immediate recovery area. Patients underwent same-day discharge as per standard protocol or were admitted to hospital if necessary or requested by the proceduralist.

### Procedural characteristics

Procedural characteristics collected included lesion sets (e.g. PVI, PW isolation, superior vena cava isolation), total number of PFA applications including to the PVs and the PW, total procedure time, left atrial catheter dwell time, fluoroscopy time and radiation dose by air kerma (mGy) and dose-area product (µGym^2^).

### Adverse events

Adverse events evaluated at 30 days post-procedure were classified as either major or minor. Major adverse events were defined as those resulting in death, permanent injury, the need for significant intervention, or presenting a life-threatening risk. These included atrio-oesophageal fistula, symptomatic pulmonary vein (PV) stenosis, cardiac tamponade, stroke, coronary artery spasm, persistent phrenic nerve injury, major vascular complications requiring surgical intervention, and the need for dialysis. Minor adverse events were defined as self-limiting events or those not requiring surgical intervention. These included systemic air embolism, transient ischaemic attack, transient phrenic nerve injury, minor vascular complications requiring non-surgical intervention (such as FemoStop™ application or additional manual compression), pericarditis, pulmonary oedema, haemolysis, and transient renal impairment (defined as a post-procedure doubling of serum creatinine compared with baseline). For patients admitted following the procedure, day-one post-procedure renal function data were collected when available. Routine follow-up phone calls were conducted by nursing staff seven days after the procedure, and patients were subsequently reviewed either in the tertiary centre arrhythmia clinic or by their private cardiologist.

### Statistical analysis

Categorical variables are presented as frequencies and percentages. Continuous variables are reported as mean with standard deviation if there was reasonably symmetrical distribution on visual inspection of histogram or otherwise reported as median with interquartile range (IQR). Patients were stratified by type of ablation system used for comparative analysis. Between-group comparisons were performed using Pearson’s chi-square test for categorical variables. For continuous variables, comparison between two groups was performed using t-test. Comparison between three groups was performed using analysis of variance (ANOVA) with a subsequent analysis of normality of residuals visually using histograms and Q-Q plots. Homogeneity of variance was also assessed. Where either of these assumptions were violated, Kruskal-Wallis test was performed instead. All analyses were performed using Stata MP 19.0 (StataCorp LP, College Station, TX, United States).

## Results

### Baseline characteristics

A total of 150 patients were included with 50 consecutive patients undergoing PFA with Farawave, 50 consecutive patients undergoing PFA with Faraview and 50 consecutive patients undergoing PFA with Varipulse (Table 1). The median age was 63–65 years (*p* = 0.28 between groups) and 28–38% patients being female (*p* = 0.60). Mean BMI was between 28.6 and 29.9 kg/m^2^ (*p* = 0.43) with a median CHA_2_DS_2_-VA of 2 (IQR 1–3; *p* = 0.96). There was no significant difference between groups with regards to cardiovascular comorbidities. Persistent AF ranged from 40 to 62% which was not statistically significant between groups (*p* = 0.09). Median LVEF was 55–60% (*p* = 0.30) and LAVI 43–49 ml/m^2^ (*p* = 0.47).

**Table 1:** Patient characteristics

	Farawave *N* = 50	Faraview *N* = 50	Varipulse *N* = 50	*p*-value
Age, years	63 (56–69)	65 (57–72)	64 (58–72)	0.28
Female (%)	19 (38)	17 (34)	14 (28)	0.60
BMI, kg/m^2^ (mean ± SD)	28.6 ± 5.2	29.4 ± 5.2	29.9 ± 4.7	0.43
CHA₂DS₂-VA, median (IQR)	2 (1–3)	2 (1–3)	2 (1–2)	0.96
Comorbidities (%)				
Hypertension	28 (56)	25 (50)	26 (52)	0.94
Vascular disease	8 (16)	9 (18)	8 (16)	0.93
Heart failure	10 (20)	14 (28)	15 (30)	0.50
Diabetes mellitus	6 (12)	4 (8)	7 (14)	0.73
Stroke/TIA	6 (12)	2 (4)	4 (8)	0.39
eGFR, median (IQR)	76 (69–90)	79 (62–90)	86 (71–90)	0.55
Type of AF (%)				
Paroxysmal	30 (60)	25 (50)	19 (38)	0.094
Persistent	20 (40)	25 (50)	31 (62)	
LVEF, % median (IQR)	60 (53–60)	60 (50–65)	55 (40–60)	0.30
LAVI, ml/m^2^	43 (36–53)	49 (40–54)	44 (40–52)	0.47
Antiarrhythmic drug				
None	12 (24)	20 (40)	14 (28)	0.13
Flecainide	10 (20)	12 (25)	7 (14)	
Sotalol	19 (38)	13 (26)	15 (30)	
Amiodarone	9 (18)	5 (10)	14 (28)	
Anticoagulation prior				
None	3 (6)	7 (14)	2 (4)	0.074
Apixaban	37 (74)	33 (66)	38 (76)	
Rivaroxaban	8 (16)	8 (16)	8 (16)	
Dabigatran	1 (2)	1 (2)	2 (4)	
Warfarin	1 (2)	1 (2)	0	

*AF*, atrial fibrillation; *BMI*, Body mass index; *IQR*, interquartile range; *LAVI*, left atrial volume indexed; *LVEF*, left ventricular ejection fraction; *SD*, Standard deviation; *TIA*, transient ischaemic attack

### Procedural lesions

All patients underwent PVI (Table 2). The rate of PW ablation differed between groups; 50% of patients in the Faraview group, 26% in the Varipulse group and 24% in Farawave group (*p* = 0.01). There was no significant difference between groups in the rate of superior vena cava (SVC) isolation (*p* = 0.16). The total PFA applications was significantly higher in patients undergoing Faraview compared with Farawave (median 60 versus 39 applications; *p* < 0.001) with a correspondingly higher number of PV applications (median 52 versus 36 applications; *p* < 0.001) although fewer PW applications (median 18 versus 24 applications; *p* = 0.028). The total PFA applications was 22 (IQR 20–27) in the Varipulse group. All procedures were performed successfully with loss of local intracardiac EGM signals as marker of successful isolation at time of ablation. Of note, 8 patients (16%) who underwent PFA with Varipulse also underwent cavotricuspid isthmus (CTI) ablation using a focal radiofrequency catheter during the same procedure.

**Table 2:** Procedure characteristics

	Farawave *N* = 50	Faraview *N* = 50	Varipulse *N* = 50	*p*-value
Ablation				
Pulmonary vein	50 (100)	50 (100)	50 (100)	N/A
Posterior wall	12 (24)	25 (50)	13 (26)	0.01
Superior vena cava	0	3 (6)	4 (8)	0.16
Number of PFA applications				
Total applications	39 (32-50)	60 (56-68)	22 (20-27)	N/A
Pulmonary vein applications	36 (32-44)^a^	52 (48-60)^a^	21 (19-23)	
Posterior wall applications	24 (22-24)^b^	18 (16-20)^b^	8 (6-10)	
Superior vena cava applications	N/A	4 (4-6)	3 (2-3)	
Additional lesion sets				
Cavotricuspid isthmus line	0	0	8 (16)	
Procedural times				
Procedural time, minutes	81 (63-90)	81 (67-101)	104 (93-119)	<0.001
Left atrial catheter dwell time, minutes	42 (35-58)	51 (44-65)	61 (52-71)	<0.001
Fluoroscopy time, minutes	20 (15-27)	21 (16-27)	12 (9-18)	<0.001
Radiation dose, Air Kerma mGy	176 ± 188	206 ± 112	238 ± 230	0.25
Radiation dose, Dose-area product µGym^2^	2018 ± 2021	2307 ± 1316	2350 ± 1807	0.59

*PFA*, Pulsed field ablation

^a^Statistically significant difference between Farawave and Faraview on t-test

^b^Statistically significant difference between Farawave and Faraview on t-test

### Procedure time

**Fig. 2:**
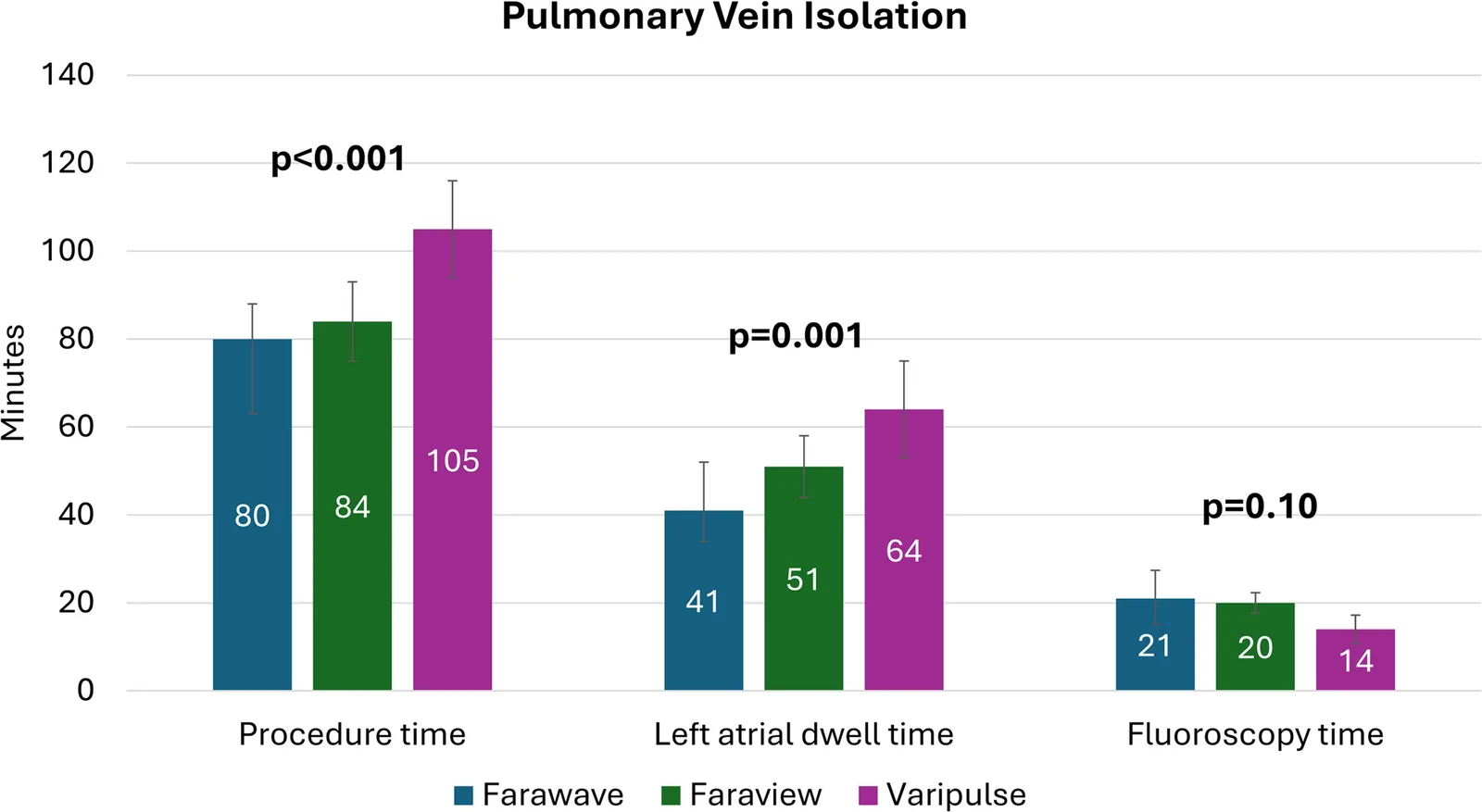
Procedure times between different pulsed field ablation platforms. Procedure time, left atrial dwell time and fluoroscopy time as it relates to patients who underwent pulmonary vein isolation only to enable comparison between PFA platforms

**Fig. 3:**
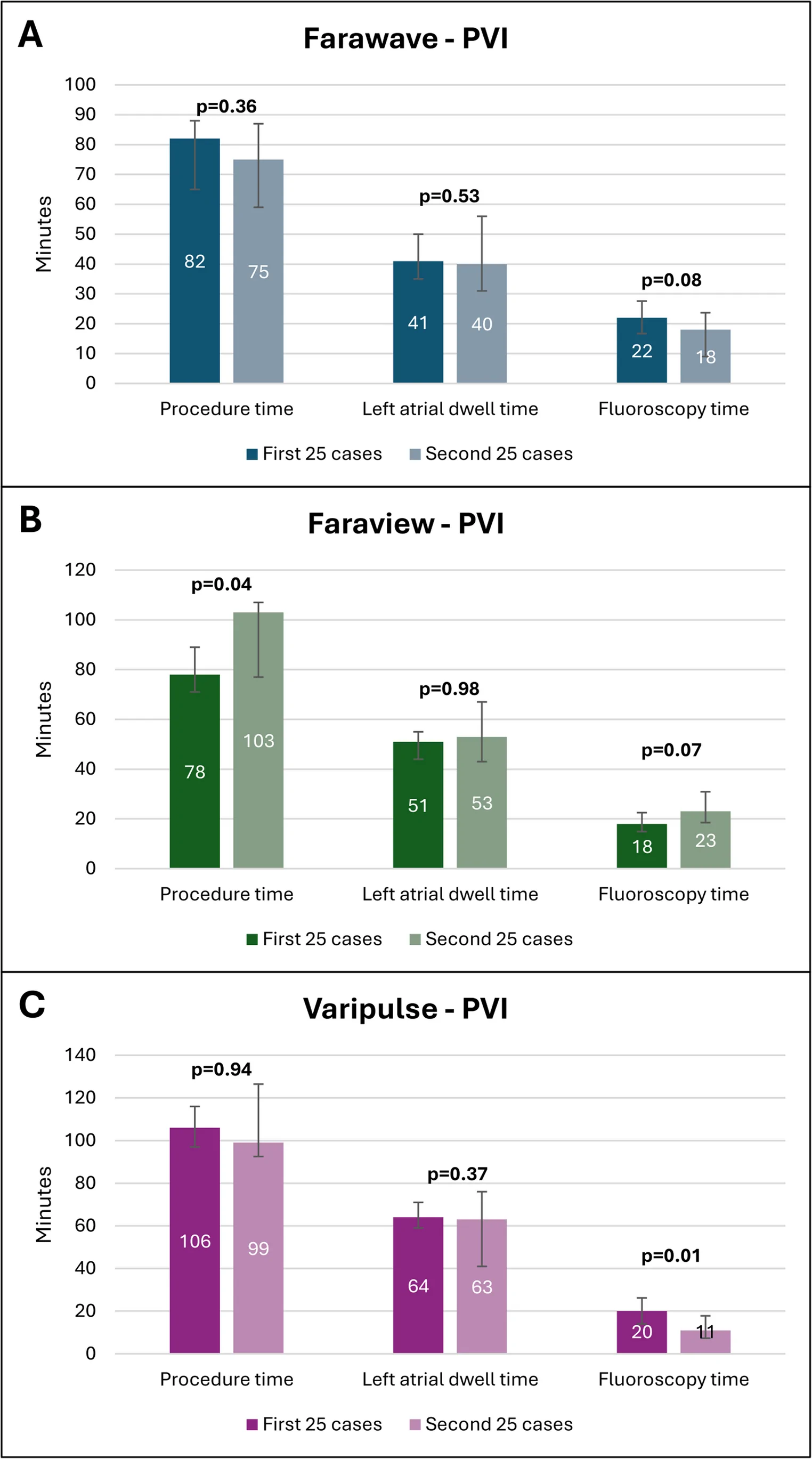
Learning curve for procedure duration for each pulsed field ablation platform. *PVI, pulmonary vein isolation*

Procedure time and left atrial catheter dwell time were increased in patients undergoing Varipulse (median 104 min and 61 min, respectively) compared with Farawave (median 81 min and 42 min, respectively) or Faraview (81 min and 51 min, respectively) which was statistically significant (*p* < 0.001; Fig. 2). However, fluoroscopy time was significantly less with Varipulse (median 12 min) compared with Farawave (median 20 min) and Faraview (median 21 min) (*p* < 0.001). There was no significant difference between groups with regards to the total radiation dose (dose-area product µGym^2^; *p* = 0.59). Comparing the first 25 cases with the second 25 cases in each group, there was no significant difference in the procedure or left atrial catheter dwell time for each PFA system (Fig. 3). However, fluoroscopy use with Varipulse was significantly reduced in the second 25 cases (median 11 min) compared with the first 25 cases (median 20 min) (*p* = 0.01).

### Adverse events

There was one (2%) major adverse event (vascular complication) in the Farawave group, one (2%) in the Faraview group (cardiac tamponade) and none in the Varipulse group with no statistically significant difference between groups (Table 3). The rate of minor adverse events was 9 (18%) for Farawave, 6 (12%) for Faraview and 4 (8%) for Varipulse which was also not statistically significant between groups (*p* = 0.36). This was predominantly minor vascular complications across the groups, which occurred in 7 (14%) of the Farawave group, 3 (6%) in the Faraview group and 3 (6%) in the Varipulse group. In the Faraview group, pulmonary oedema also occurred in 2 (4%) patients). There was one (2%) case of pericarditis in the Farawave and the Faraview arm.

**Table 3:** Adverse events at 30 days

	Farawave *N* = 50	Faraview *N* = 50	Varipulse *N* = 50	*p*-value
Major adverse events (%)	1 (2)	1 (2)	0	1
Death	0	0	0	N/A
Atrio-oesophageal fistula	0	0	0	N/A
Symptomatic pulmonary vein stenosis	0	0	0	N/A
Cardiac tamponade	0	1 (2)	0	1
Stroke	0	0	0	N/A
Coronary artery spasm	0	0	0	N/A
Persistent phrenic nerve injury	0	0	0	N/A
Major vascular complication	1 (2)	0	0	1
Dialysis requirement	0	0	0	N/A
Minor adverse events (%)	9 (18)	6 (12)	4 (8)	0.36
Systemic air embolism	0	0	0	N/A
Transient ischaemic attack	1 (2)	1 (2)	0	1
Transient phrenic nerve injury	0	0	0	N/A
Minor vascular complication	7 (14)	3 (6)	3 (6)	0.30
Pericarditis	1 (2)	1 (2)	0	1
Pulmonary oedema	0	2 (4)	0	0.33
Haemolysis	0	0	0	N/A
Transient renal impairment	0	1 (2)	0	1

## Discussion

With an increasing number of PFA systems emerging from industry, there is a need to understand their real-world application and individual procedural workflow. We thus analysed procedural characteristics and acute outcomes in patients undergoing PFA using the Farapulse system, Faraview system and Varipulse system at our centre. In consecutive patients with no significant differences in baseline demographics and comorbidities, we found that

1. PW ablation was more frequently performed with Faraview compared with Farawave or Varipulse despite no significant difference in proportion of persistent AF in each group.
2. More PV applications and fewer PW applications were delivered with Faraview compared with Farawave.
3. Procedure and left atrial catheter dwell time was higher while fluoroscopy time was less with Varipulse compared with Farawave and Faraview.
4. There was no difference in major or minor adverse events between PFA systems.

There were significant differences in the rate of PW ablation and the number of PV and PW PFA applications performed with the integrated use of 3D mapping in the Faraview system compared with Farawave (performed under fluoroscopy and TOE guidance alone). There is an inverse association between the distance between wide area circumferential ablation for PVI and the risk of roof-dependent atrial flutter [8]. As such, there is concern that a potential residual narrow channel on the PW could predispose to roof-dependent atrial flutter and this has been observed even after PFA in patients with reconnections in the PW [9]. The integration of EAM with Faraview enables additional visualisation of left atrium geometry and enables recognition of previous PFA lesion coverage and potential gaps using their Fieldtag™ technology. This potentially leads to increased recognition of a perceived narrow channel on the PW resulting in preferentially ablating the PW as well. It is notable that there were also greater PV applications with Faraview compared with Farawave which might suggest that the addition of the EAM guides areas of previous ablation and identifies further potential gaps in achieving full isolation. Similarly, EAM may also mitigate potentially unnecessary additional lesions as observed in the fewer number of PW applications with Faraview compared with Farawave.

Given the relative ease and rapidity in delivering PFA lesions with single-shot technology, there may be a propensity towards an increased number of applications either for PVs, carina or non-PV triggers such as the posterior wall or SVC. In the MANIFEST-US multi-centre registry using the pentaspline catheter [10], 57% of patients received PW ablation despite the present lack of randomised data to support this ablation strategy in reducing arrhythmia recurrence [11, 12]. The integration of EAM to the pentaspline catheter with Faraview makes it even procedurally easier to ablate the PW compared with Farapulse under fluoroscopy alone. However, procedural convenience is not a rationale alone for additional ablation and it is well-established that increased PFA applications confers an increased risk of haemolysis and unnecessarily increases procedural duration [13]. It is important that there remains a strong evidence base to justify what we do procedurally on patients and we await more robust data including randomised trials to clarify the role of PFA for potential non-PV triggers prior to routine adoption in clinical practice.

Varipulse was associated with increased overall procedure time and left atrial catheter dwell time compared with Farapulse and Faraview. The reasons for the increased procedure time are likely multifactorial. First, eight patients who underwent Varipulse PFA also underwent a CTI line with a radiofrequency catheter that could be visualised and integrated on the existing Carto system. Second, the availability of full high-fidelity integrated EAM enables operators to undertake a thorough pre- and post-ablation map. Conversely, with availability of high-fidelity EAM, operators are likely less reliant on additional fluoroscopy imaging as was observed with the reduced fluoroscopy time with Varipulse compared with Farawave or Faraview. Interestingly, there was a minor learning curve with Varipulse observed with reduced fluoroscopy time in the second 25 cases compared with the first 25 cases. Furthermore, the Varipulse system also provides information on catheter contact based on TPI for each electrode. This is intended to lead to optimisation of catheter position to improve catheter-tissue apposition and will tend to increase procedure time. A small Japanese study has shown that PV reconnection is significantly higher in patients with lower TPI-positive areas and such data ultimately highlights the importance of catheter-tissue contact [7]. Improvements in catheter design and procedural technique are needed given current clinical data suggest PV and PW reconnection rates beyond 50% in patients with symptomatic arrhythmia recurrences undergoing repeat procedure after index PFA [14].

There were no significant differences in major or minor adverse events between PFA systems in this study although significant differences are unlikely to be detected with our sample size. Post-approval registry from the United States in over 40,000 patients undergoing PFA with the Farapulse system has reported a major adverse event rate of 0.63% including death at 30 days of 0.04%, cardiac tamponade in 0.16% and vascular injury requiring intervention of 0.18% [15]. However, it is notable that minor vascular access complications were numerically higher with the Farawave system compared with Faraview or Varipulse in our analysis. While reporting of minor vascular access complications is likely variable between studies depending on how it is defined, the sheath size (16.8Fr outer) with the Faradrive/Farapulse is larger than the Vizigo sheath (11.5Fr outer) that was used with the Varipulse system. A randomised trial has shown that the use of a vascular closure device with the Farapulse system significantly reduced time to ambulation and minor bleeding complications compared with a standard figure-of-eight suture [16]. Nevertheless, numerous large registry data have shown an acceptable safety profile with the Farapulse system and further data are awaited with Varipulse [5, 17, 18].

### Limitations

We acknowledge several limitations in this analysis. First, this was a single tertiary centre observational study and is biased based on operator preference in terms of choice of PFA platform and their individual procedural workflow. Second, this analysis is limited to procedural workflow and procedural outcomes at short-term follow-up; additional long-term follow-up data are needed to assess arrhythmia recurrence. Additionally, this study is not powered to detect differences in rarer adverse events. Third, the Varipulse platform was introduced at a later stage compared with the Farapulse/Faraview system. This could influence operator familiarity with PFA technology although it is notable that the procedure and left atrial dwell time were higher with Varipulse. Fourth, our centre does not routinely assess for subclinical haemolysis in the form of serum haemolysis markers and routinely practices same-day discharge. Thus, the risk of acute kidney injury may be under-estimated. Nevertheless, the clinical sequelae of subclinical haemolysis appears limited with judicious use of PFA in combination with improved catheter-tissue contact.

## Conclusions

In this tertiary centre analysis of procedural characteristics and outcomes in patients undergoing PFA with different systems, PW ablation was more commonly performed in the Faraview group. Use of Faraview system was associated with more PV applications but fewer PW applications compared with the Farapulse system. Varipulse was associated with an increased procedure time although reduced fluoroscopy time compared with Farapulse and Faraview systems. There were no detected differences in the rate of major or minor adverse events albeit with a small sample size. These real-world data provide insight into the adaptation in procedural workflow based on PFA catheter design and the role of EAM and tissue contact information.

## Data Availability

No datasets were generated or analysed during the current study.
